# Crystal structure of difenoconazole

**DOI:** 10.1107/S1600536814022429

**Published:** 2014-10-24

**Authors:** Seonghwa Cho, Gihaeng Kang, Sangjin Lee, Tae Ho Kim

**Affiliations:** a Department of Chemistry and Research Institute of Natural Sciences, Gyeongsang National University, Jinju 660-701, Republic of Korea

**Keywords:** crystal structure, difenoconazole, triazole fungicide, hydrogen bonds

## Abstract

In the title compound difenoconazole [systematic name: 1-({2-[2-chloro-4-(4-chloro­phen­oxy)phen­yl]-4-methyl-1,3-dioxolan-2-yl}meth­yl)-1*H*-1,2,4-triazole], C_19_H_17_Cl_2_N_3_O_3_, the dihedral angle between the planes of the 4-chloro­phenyl and 2-chloro­phenyl rings is 79.34 (9)°, while the dihedral angle between the planes of the triazole ring and the dioxolanyl group is 59.45 (19)°. In the crystal, pairs of C—H⋯N hydrogen bonds link adjacent mol­ecules, forming dimers with *R*_2_^2^(6) loops. In addition, the dimers are linked by C—H⋯O hydrogen bonds, resulting in a three-dimensional architecture. Disorder was modeled for one C atom of the dioxolanyl group over two sets of sites with an occupancy ratio of 0.566 (17):0.434 (17).

## Related literature   

For information on the toxicity and fungicidal properties of the title compound, see: Dong *et al.* (2013 ▶); Mu *et al.* (2013 ▶). For a related crystal structure, see: Jeon *et al.* (2013 ▶).
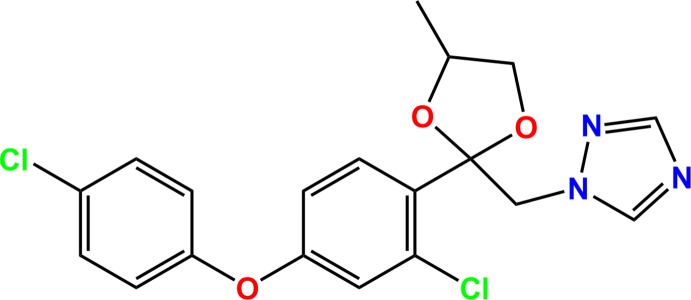


## Experimental   

### Crystal data  

C_19_H_17_Cl_2_N_3_O_3_
*M* *_r_* = 406.26
Monoclinic, 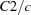

*a* = 25.6913 (13) Å
*b* = 10.0220 (4) Å
*c* = 14.6417 (6) Å
β = 93.439 (4)°
*V* = 3763.1 (3) Å^3^
*Z* = 8
Mo *K*α radiation
μ = 0.37 mm^−1^
*T* = 173 K
0.50 × 0.42 × 0.34 mm

### Data collection  

Bruker APEXII CCD diffractometer
Absorption correction: multi-scan (*SADABS*; Bruker, 2009 ▶) *T*_min_ = 0.837, *T*_max_ = 0.885
28161 measured reflections
3694 independent reflections
3251 reflections with *I* > 2σ(*I*)
*R*_int_ = 0.040

### Refinement  

*R*[*F* ^2^ > 2σ(*F* ^2^)] = 0.059
*wR*(*F* ^2^) = 0.160
*S* = 1.06
3694 reflections
254 parameters
H-atom parameters constrained
Δρ_max_ = 1.08 e Å^−3^
Δρ_min_ = −0.82 e Å^−3^

### 

Data collection: *APEX2* (Bruker, 2009 ▶); cell refinement: *SAINT* (Bruker, 2009 ▶); data reduction: *SAINT*; program(s) used to solve structure: *SHELXTL* (Sheldrick, 2008 ▶); program(s) used to refine structure: *SHELXTL*; molecular graphics: *DIAMOND* (Brandenburg, 2010 ▶); software used to prepare material for publication: *SHELXTL*.

## Supplementary Material

Crystal structure: contains datablock(s) global, I. DOI: 10.1107/S1600536814022429/hg5412sup1.cif

Structure factors: contains datablock(s) I. DOI: 10.1107/S1600536814022429/hg5412Isup2.hkl

Supporting information file. DOI: 10.1107/S1600536814022429/hg5412Isup3.cml

**Fig. 1.**
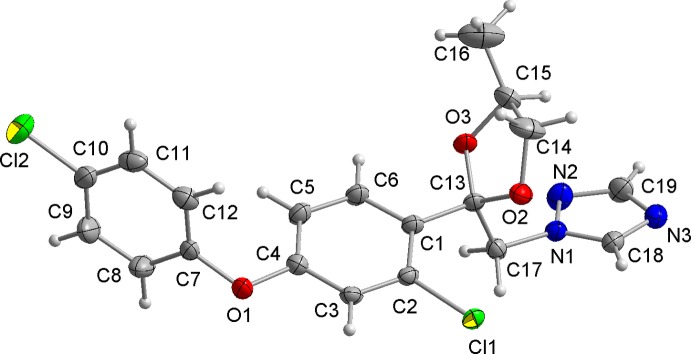
The asymmetric unit of the title compound with the atom numbering scheme. Displacement ellipsoids are drawn at the 50% probability level. H atoms are shown as small spheres of arbitrary radius. Only atoms of the major disorder components are shown.

. DOI: 10.1107/S1600536814022429/hg5412fig1.tif
The asymmetric unit of the title compound with the atom numbering scheme. Displacement ellipsoids are drawn at the 50% probability level. H atoms are shown as small spheres of arbitrary radius. Only atoms of the major disorder components are shown.

**Fig. 2.**
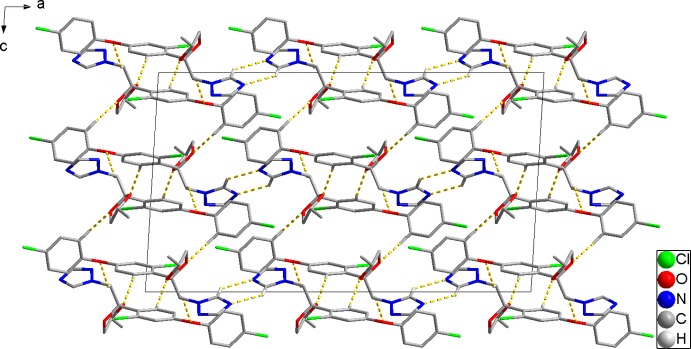
Crystal packing of the title compound with C—H···N and C—H···O hydrogen bonds are shown as dashed lines. H atoms bonded to C atoms have been omitted for clarity, except H atoms of hydrogen bonds. Only atoms of the major disorder components are shown.

. DOI: 10.1107/S1600536814022429/hg5412fig2.tif
Crystal packing of the title compound with C—H⋯N and C—H⋯O hydrogen bonds are shown as dashed lines. H atoms bonded to C atoms have been omitted for clarity, except H atoms of hydrogen bonds. Only atoms of the major disorder components are shown.

CCDC reference: 1028719

Additional supporting information:  crystallographic information; 3D view; checkCIF report

## Figures and Tables

**Table 1:** Hydrogen-bond geometry (, )

*D*H*A*	*D*H	H*A*	*D* *A*	*D*H*A*
C6H6O3^i^	0.95	2.48	3.314(3)	146
C12H12O2^ii^	0.95	2.40	3.226(3)	145
C17H17*A*O1^iii^	0.99	2.58	3.378(3)	138
C18H18N3^iv^	0.95	2.57	3.323(4)	136

Symmetry codes: (i) 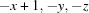; (ii) 
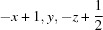
; (iii) 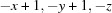; (iv) 
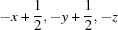
.

## supplementary crystallographic information

### S1. Comment

Difenoconazole, C_19_H_17_Cl_2_N_3_O_3_, is a member of the triazoles group of fungicides and it has been used for the control of fungal diseases on fruits, vegetables, cereals, and other field crops (Dong *et al.*, 2013; Mu *et al.*, 2013). The structure of this compound is reported herein. In this compound (Fig. 1), the dihedral angle between the 4-chlorophenyl ring and the 2-chlorophenyl ring is 79.34 (9)°, while the dihedral angle between the triazole ring and dioxolanyl group plane is 59.45 (19)°. Disorder was modeled for one C atom (C15) of the dioxolanyl group group over two sets of sites with an occupancy ratio of 0.566 (17):0.434 (17). All bond lengths and bond angles are normal and comparable to those observed in the crystal structure of a similar compound (Jeon *et al.*, 2013).

In the crystal lattice (Fig. 2, Table 1), two C18—H18···N3 hydrogen bonds link adjacent molecules, forming dimers with *R*_2_^2^(6) loops. In addition, the dimers linked by C6—H6···O3, C12—H12···O2, and C17—H17A···O1 hydrogen bonds, resulting in a three-dimensional architecture.

### S2. Experimental

The title compound was purchased from the Dr. Ehrenstorfer GmbH Company. Slow evaporation of a solution in CH_2_Cl_2_ gave single crystals suitable for X-ray analysis.

### S3. Refinement

During refinement, the C15 atom of the dioxolanyl group was disordered and was refined using a split model. The corresponding site-occupation factors were refined so that their sum was unity [0.566 (17) and 0.434 (17)]. All H-atoms were positioned geometrically and refined using a riding model with d(C—H) = 0.98 Å, *U*_iso_ = 1.5*U*_eq_(C) for methyl group, d(C—H) = 0.99 Å, *U*_iso_ = 1.2*U*_eq_(C) for C*sp*^3^—H, d(C—H) = 0.95 Å, *U*_iso_ = 1.2*U*_eq_(C) for aromatic C—H, and d(C—H) = 1.00 Å, *U*_iso_ = 1.5*U*_eq_(C) for C*sp*^3^—H.

### Crystal data

**Table:**

C_19_H_17_Cl_2_N_3_O_3_	*F*(000) = 1680
*M**_r_* = 406.26	*D*_x_ = 1.434 Mg m^−^^3^
Monoclinic, *C*2/*c*	Mo *K*α radiation, λ = 0.71073 Å
Hall symbol: -C 2yc	Cell parameters from 9891 reflections
*a* = 25.6913 (13) Å	θ = 2.2–27.5°
*b* = 10.0220 (4) Å	µ = 0.37 mm^−^^1^
*c* = 14.6417 (6) Å	*T* = 173 K
β = 93.439 (4)°	Block, colourless
*V* = 3763.1 (3) Å^3^	0.50 × 0.42 × 0.34 mm
*Z* = 8	

### Data collection

**Table:**

Bruker APEXII CCD diffractometer	3694 independent reflections
Radiation source: fine-focus sealed tube	3251 reflections with *I* > 2σ(*I*)
Graphite monochromator	*R*_int_ = 0.040
φ and ω scans	θ_max_ = 26.0°, θ_min_ = 1.6°
Absorption correction: multi-scan (*SADABS*; Bruker, 2009)	*h* = −31→31
*T*_min_ = 0.837, *T*_max_ = 0.885	*k* = −12→11
28161 measured reflections	*l* = −16→18

### Refinement

**Table:**

Refinement on *F*^2^	Primary atom site location: structure-invariant direct methods
Least-squares matrix: full	Secondary atom site location: difference Fourier map
*R*[*F*^2^ > 2σ(*F*^2^)] = 0.059	Hydrogen site location: inferred from neighbouring sites
*wR*(*F*^2^) = 0.160	H-atom parameters constrained
*S* = 1.06	*w* = 1/[σ^2^(*F*_o_^2^) + (0.0826*P*)^2^ + 11.4923*P*] where *P* = (*F*_o_^2^ + 2*F*_c_^2^)/3
3694 reflections	(Δ/σ)_max_ = 0.001
254 parameters	Δρ_max_ = 1.08 e Å^−^^3^
0 restraints	Δρ_min_ = −0.82 e Å^−^^3^

### Special details

**Table:**

Geometry. All e.s.d.'s (except the e.s.d. in the dihedral angle between two l.s. planes) are estimated using the full covariance matrix. The cell e.s.d.'s are taken into account individually in the estimation of e.s.d.'s in distances, angles and torsion angles; correlations between e.s.d.'s in cell parameters are only used when they are defined by crystal symmetry. An approximate (isotropic) treatment of cell e.s.d.'s is used for estimating e.s.d.'s involving l.s. planes.
Refinement. Refinement of *F*^2^ against ALL reflections. The weighted *R*-factor *wR* and goodness of fit *S* are based on *F*^2^, conventional *R*-factors *R* are based on *F*, with *F* set to zero for negative *F*^2^. The threshold expression of *F*^2^ > σ(*F*^2^) is used only for calculating *R*-factors(gt) *etc*. and is not relevant to the choice of reflections for refinement. *R*-factors based on *F*^2^ are statistically about twice as large as those based on *F*, and *R*- factors based on ALL data will be even larger.

### Fractional atomic coordinates and isotropic or equivalent isotropic displacement parameters (Å^2^)

**Table:**

	*x*	*y*	*z*	*U*_iso_*/*U*_eq_	Occ. (<1)
Cl1	0.42744 (2)	0.49115 (6)	0.12241 (5)	0.0266 (2)	
Cl2	0.82170 (3)	0.20023 (10)	0.20260 (9)	0.0669 (4)	
O1	0.62242 (7)	0.48045 (19)	0.13830 (15)	0.0310 (5)	
O2	0.39803 (7)	0.19880 (19)	0.13311 (13)	0.0278 (4)	
O3	0.44578 (7)	0.04892 (18)	0.05807 (14)	0.0310 (5)	
N1	0.35985 (8)	0.1646 (2)	−0.04986 (15)	0.0238 (5)	
N2	0.35747 (10)	0.0522 (3)	−0.10262 (18)	0.0351 (6)	
N3	0.27774 (9)	0.1059 (2)	−0.05524 (16)	0.0311 (5)	
C1	0.48411 (10)	0.2656 (3)	0.08715 (18)	0.0234 (5)	
C2	0.48477 (10)	0.4006 (2)	0.11281 (17)	0.0217 (5)	
C3	0.53104 (10)	0.4691 (3)	0.13124 (17)	0.0232 (5)	
H3	0.5305	0.5600	0.1496	0.028*	
C4	0.57807 (10)	0.4041 (3)	0.12269 (18)	0.0241 (5)	
C5	0.57897 (10)	0.2708 (3)	0.09724 (19)	0.0280 (6)	
H5	0.6112	0.2260	0.0917	0.034*	
C6	0.53236 (10)	0.2042 (3)	0.08011 (19)	0.0274 (6)	
H6	0.5332	0.1129	0.0628	0.033*	
C7	0.66965 (10)	0.4125 (3)	0.15259 (19)	0.0246 (5)	
C8	0.70587 (12)	0.4212 (3)	0.0878 (2)	0.0368 (7)	
H8	0.6985	0.4704	0.0331	0.044*	
C9	0.75346 (12)	0.3571 (3)	0.1033 (3)	0.0446 (8)	
H9	0.7792	0.3625	0.0596	0.054*	
C10	0.76287 (11)	0.2854 (3)	0.1831 (2)	0.0372 (7)	
C11	0.72675 (12)	0.2774 (3)	0.2478 (2)	0.0337 (7)	
H11	0.7339	0.2273	0.3022	0.040*	
C12	0.67983 (11)	0.3430 (3)	0.23296 (19)	0.0308 (6)	
H12	0.6547	0.3404	0.2779	0.037*	
C13	0.43413 (10)	0.1863 (2)	0.06428 (18)	0.0235 (5)	
C14	0.39958 (15)	0.0777 (3)	0.1848 (2)	0.0465 (8)	
H14A	0.3646	0.0559	0.2053	0.056*	0.566 (17)
H14B	0.4241	0.0860	0.2393	0.056*	0.566 (17)
H14C	0.4047	0.0970	0.2510	0.056*	0.434 (17)
H14D	0.3666	0.0274	0.1738	0.056*	0.434 (17)
C15	0.4172 (4)	−0.0259 (7)	0.1230 (6)	0.042 (3)	0.566 (17)
H15	0.3851	−0.0594	0.0883	0.064*	0.566 (17)
C15'	0.4417 (4)	0.0044 (8)	0.1547 (6)	0.040 (3)	0.434 (17)
H15'	0.4736	0.0383	0.1899	0.059*	0.434 (17)
C16	0.4442 (2)	−0.1428 (4)	0.1591 (3)	0.0714 (13)	
H16A	0.4771	−0.1534	0.1293	0.107*	0.566 (17)
H16B	0.4223	−0.2218	0.1472	0.107*	0.566 (17)
H16C	0.4514	−0.1325	0.2252	0.107*	0.566 (17)
H16D	0.4503	−0.1784	0.0983	0.107*	0.434 (17)
H16E	0.4111	−0.1777	0.1792	0.107*	0.434 (17)
H16F	0.4727	−0.1699	0.2025	0.107*	0.434 (17)
C17	0.40879 (10)	0.2322 (3)	−0.02759 (18)	0.0245 (5)	
H17A	0.4026	0.3296	−0.0254	0.029*	
H17B	0.4330	0.2148	−0.0764	0.029*	
C18	0.31254 (10)	0.1939 (3)	−0.02307 (18)	0.0248 (5)	
H18	0.3048	0.2680	0.0142	0.030*	
C19	0.30749 (12)	0.0227 (3)	−0.1031 (2)	0.0357 (7)	
H19	0.2932	−0.0524	−0.1351	0.043*	

### Atomic displacement parameters (Å^2^)

**Table:**

	*U*^11^	*U*^22^	*U*^33^	*U*^12^	*U*^13^	*U*^23^
Cl1	0.0242 (3)	0.0184 (3)	0.0374 (4)	0.0054 (2)	0.0048 (3)	−0.0025 (2)
Cl2	0.0336 (5)	0.0520 (6)	0.1140 (9)	0.0139 (4)	−0.0048 (5)	0.0196 (5)
O1	0.0219 (9)	0.0197 (10)	0.0513 (13)	−0.0017 (7)	0.0017 (8)	0.0011 (8)
O2	0.0288 (10)	0.0241 (10)	0.0310 (10)	0.0005 (8)	0.0061 (8)	0.0041 (7)
O3	0.0271 (10)	0.0141 (9)	0.0518 (13)	0.0024 (7)	0.0020 (8)	−0.0014 (8)
N1	0.0232 (11)	0.0182 (11)	0.0298 (12)	0.0005 (8)	0.0003 (8)	−0.0026 (8)
N2	0.0320 (13)	0.0267 (13)	0.0462 (15)	0.0009 (10)	0.0000 (11)	−0.0134 (11)
N3	0.0256 (12)	0.0322 (13)	0.0350 (13)	−0.0012 (10)	−0.0010 (9)	0.0043 (10)
C1	0.0239 (13)	0.0178 (12)	0.0283 (13)	0.0020 (10)	0.0007 (10)	0.0001 (10)
C2	0.0234 (12)	0.0173 (12)	0.0247 (13)	0.0045 (10)	0.0039 (10)	0.0013 (9)
C3	0.0271 (13)	0.0150 (12)	0.0278 (13)	0.0011 (10)	0.0036 (10)	0.0015 (10)
C4	0.0231 (13)	0.0210 (13)	0.0280 (13)	−0.0014 (10)	0.0013 (10)	0.0025 (10)
C5	0.0240 (13)	0.0230 (14)	0.0370 (15)	0.0055 (10)	0.0004 (11)	−0.0014 (11)
C6	0.0261 (13)	0.0181 (13)	0.0377 (15)	0.0032 (10)	0.0000 (11)	−0.0027 (10)
C7	0.0193 (12)	0.0183 (12)	0.0359 (14)	−0.0016 (10)	0.0004 (10)	−0.0001 (10)
C8	0.0359 (16)	0.0347 (16)	0.0406 (17)	0.0062 (13)	0.0089 (13)	0.0149 (13)
C9	0.0311 (16)	0.0447 (19)	0.060 (2)	0.0059 (14)	0.0187 (14)	0.0165 (16)
C10	0.0263 (14)	0.0254 (15)	0.059 (2)	0.0010 (11)	−0.0044 (13)	0.0050 (13)
C11	0.0417 (16)	0.0247 (14)	0.0331 (15)	−0.0042 (12)	−0.0099 (12)	0.0021 (11)
C12	0.0369 (15)	0.0255 (14)	0.0303 (14)	−0.0037 (12)	0.0047 (11)	−0.0011 (11)
C13	0.0236 (12)	0.0135 (12)	0.0336 (14)	0.0032 (10)	0.0036 (10)	−0.0013 (10)
C14	0.070 (2)	0.0269 (16)	0.0443 (19)	−0.0014 (16)	0.0185 (16)	0.0100 (13)
C15	0.044 (5)	0.025 (3)	0.059 (5)	−0.004 (3)	0.011 (4)	0.009 (3)
C15'	0.040 (5)	0.023 (4)	0.052 (5)	−0.012 (3)	−0.023 (4)	0.007 (3)
C16	0.106 (4)	0.035 (2)	0.074 (3)	0.006 (2)	0.008 (3)	0.0103 (19)
C17	0.0224 (12)	0.0213 (13)	0.0300 (14)	−0.0023 (10)	0.0038 (10)	−0.0015 (10)
C18	0.0233 (12)	0.0229 (13)	0.0281 (13)	0.0023 (10)	0.0012 (10)	0.0018 (10)
C19	0.0316 (15)	0.0247 (15)	0.0497 (18)	−0.0032 (12)	−0.0065 (13)	−0.0059 (12)

### Geometric parameters (Å, º)

**Table:**

Cl1—C2	1.743 (2)	C8—H8	0.9500
Cl2—C10	1.744 (3)	C9—C10	1.380 (5)
O1—C4	1.380 (3)	C9—H9	0.9500
O1—C7	1.396 (3)	C10—C11	1.367 (5)
O2—C13	1.416 (3)	C11—C12	1.379 (4)
O2—C14	1.429 (4)	C11—H11	0.9500
O3—C13	1.413 (3)	C12—H12	0.9500
O3—C15	1.445 (6)	C13—C17	1.530 (4)
O3—C15'	1.493 (9)	C14—C15'	1.401 (10)
N1—C18	1.332 (3)	C14—C15	1.467 (8)
N1—N2	1.365 (3)	C14—H14A	0.9900
N1—C17	1.448 (3)	C14—H14B	0.9900
N2—C19	1.317 (4)	C14—H14C	0.9900
N3—C18	1.322 (4)	C14—H14D	0.9900
N3—C19	1.354 (4)	C15—C16	1.445 (8)
C1—C6	1.394 (4)	C15—H15	1.0000
C1—C2	1.404 (4)	C15'—C16	1.478 (9)
C1—C13	1.530 (3)	C15'—H15'	1.0000
C2—C3	1.385 (4)	C16—H16A	0.9800
C3—C4	1.385 (4)	C16—H16B	0.9800
C3—H3	0.9500	C16—H16C	0.9800
C4—C5	1.387 (4)	C16—H16D	0.9800
C5—C6	1.381 (4)	C16—H16E	0.9800
C5—H5	0.9500	C16—H16F	0.9800
C6—H6	0.9500	C17—H17A	0.9900
C7—C8	1.371 (4)	C17—H17B	0.9900
C7—C12	1.379 (4)	C18—H18	0.9500
C8—C9	1.388 (4)	C19—H19	0.9500
			
C4—O1—C7	117.1 (2)	O2—C14—H14C	110.5
C13—O2—C14	107.6 (2)	C15—C14—H14C	135.6
C13—O3—C15	110.2 (3)	H14A—C14—H14C	79.4
C13—O3—C15'	101.7 (4)	H14B—C14—H14C	32.2
C15—O3—C15'	32.3 (3)	C15'—C14—H14D	110.5
C18—N1—N2	109.7 (2)	O2—C14—H14D	110.5
C18—N1—C17	128.8 (2)	C15—C14—H14D	80.3
N2—N1—C17	121.5 (2)	H14A—C14—H14D	32.1
C19—N2—N1	101.5 (2)	H14B—C14—H14D	132.1
C18—N3—C19	101.8 (2)	H14C—C14—H14D	108.7
C6—C1—C2	116.7 (2)	O3—C15—C16	114.2 (5)
C6—C1—C13	119.5 (2)	O3—C15—C14	103.1 (5)
C2—C1—C13	123.8 (2)	C16—C15—C14	120.5 (7)
C3—C2—C1	121.8 (2)	O3—C15—H15	106.0
C3—C2—Cl1	116.47 (19)	C16—C15—H15	106.0
C1—C2—Cl1	121.8 (2)	C14—C15—H15	106.0
C2—C3—C4	119.5 (2)	C14—C15'—C16	122.8 (8)
C2—C3—H3	120.3	C14—C15'—O3	103.9 (5)
C4—C3—H3	120.3	C16—C15'—O3	109.5 (6)
O1—C4—C3	116.1 (2)	C14—C15'—H15'	106.5
O1—C4—C5	123.5 (2)	C16—C15'—H15'	106.5
C3—C4—C5	120.4 (2)	O3—C15'—H15'	106.5
C6—C5—C4	119.1 (2)	C15—C16—C15'	32.5 (3)
C6—C5—H5	120.5	C15—C16—H16A	109.5
C4—C5—H5	120.5	C15'—C16—H16A	97.2
C5—C6—C1	122.6 (2)	C15—C16—H16B	109.5
C5—C6—H6	118.7	C15'—C16—H16B	140.9
C1—C6—H6	118.7	H16A—C16—H16B	109.5
C8—C7—C12	121.4 (3)	C15—C16—H16C	109.5
C8—C7—O1	119.0 (2)	C15'—C16—H16C	86.7
C12—C7—O1	119.5 (2)	H16A—C16—H16C	109.5
C7—C8—C9	119.0 (3)	H16B—C16—H16C	109.5
C7—C8—H8	120.5	C15—C16—H16D	93.6
C9—C8—H8	120.5	C15'—C16—H16D	109.5
C10—C9—C8	119.2 (3)	H16A—C16—H16D	50.7
C10—C9—H9	120.4	H16B—C16—H16D	70.5
C8—C9—H9	120.4	H16C—C16—H16D	154.7
C11—C10—C9	121.7 (3)	C15—C16—H16E	89.7
C11—C10—Cl2	118.4 (2)	C15'—C16—H16E	109.5
C9—C10—Cl2	120.0 (3)	H16A—C16—H16E	151.8
C10—C11—C12	119.1 (3)	H16B—C16—H16E	42.9
C10—C11—H11	120.5	H16C—C16—H16E	81.7
C12—C11—H11	120.5	H16D—C16—H16E	109.5
C7—C12—C11	119.6 (3)	C15—C16—H16F	141.8
C7—C12—H12	120.2	C15'—C16—H16F	109.5
C11—C12—H12	120.2	H16A—C16—H16F	68.1
O3—C13—O2	106.4 (2)	H16B—C16—H16F	106.8
O3—C13—C17	108.5 (2)	H16C—C16—H16F	45.4
O2—C13—C17	109.7 (2)	H16D—C16—H16F	109.5
O3—C13—C1	110.0 (2)	H16E—C16—H16F	109.5
O2—C13—C1	112.0 (2)	N1—C17—C13	112.0 (2)
C17—C13—C1	110.1 (2)	N1—C17—H17A	109.2
C15'—C14—O2	106.0 (4)	C13—C17—H17A	109.2
C15'—C14—C15	33.0 (3)	N1—C17—H17B	109.2
O2—C14—C15	105.9 (3)	C13—C17—H17B	109.2
C15'—C14—H14A	135.5	H17A—C17—H17B	107.9
O2—C14—H14A	110.6	N3—C18—N1	111.0 (2)
C15—C14—H14A	110.6	N3—C18—H18	124.5
C15'—C14—H14B	80.2	N1—C18—H18	124.5
O2—C14—H14B	110.6	N2—C19—N3	116.1 (3)
C15—C14—H14B	110.6	N2—C19—H19	122.0
H14A—C14—H14B	108.7	N3—C19—H19	122.0
C15'—C14—H14C	110.5		
			
C18—N1—N2—C19	0.1 (3)	C6—C1—C13—O3	13.0 (3)
C17—N1—N2—C19	178.1 (2)	C2—C1—C13—O3	−169.0 (2)
C6—C1—C2—C3	−0.7 (4)	C6—C1—C13—O2	131.2 (2)
C13—C1—C2—C3	−178.8 (2)	C2—C1—C13—O2	−50.8 (3)
C6—C1—C2—Cl1	178.9 (2)	C6—C1—C13—C17	−106.5 (3)
C13—C1—C2—Cl1	0.8 (4)	C2—C1—C13—C17	71.5 (3)
C1—C2—C3—C4	1.2 (4)	C13—O2—C14—C15'	8.6 (6)
Cl1—C2—C3—C4	−178.40 (19)	C13—O2—C14—C15	−25.8 (6)
C7—O1—C4—C3	164.5 (2)	C13—O3—C15—C16	−147.7 (6)
C7—O1—C4—C5	−17.3 (4)	C15'—O3—C15—C16	−68.0 (10)
C2—C3—C4—O1	177.3 (2)	C13—O3—C15—C14	−15.2 (8)
C2—C3—C4—C5	−1.0 (4)	C15'—O3—C15—C14	64.5 (9)
O1—C4—C5—C6	−177.8 (2)	C15'—C14—C15—O3	−70.5 (8)
C3—C4—C5—C6	0.3 (4)	O2—C14—C15—O3	24.6 (8)
C4—C5—C6—C1	0.1 (4)	C15'—C14—C15—C16	58.2 (11)
C2—C1—C6—C5	0.0 (4)	O2—C14—C15—C16	153.3 (6)
C13—C1—C6—C5	178.2 (3)	O2—C14—C15'—C16	−153.1 (7)
C4—O1—C7—C8	111.7 (3)	C15—C14—C15'—C16	−58.4 (10)
C4—O1—C7—C12	−71.1 (3)	O2—C14—C15'—O3	−28.4 (8)
C12—C7—C8—C9	0.8 (5)	C15—C14—C15'—O3	66.2 (7)
O1—C7—C8—C9	178.0 (3)	C13—O3—C15'—C14	37.8 (7)
C7—C8—C9—C10	0.6 (5)	C15—O3—C15'—C14	−71.6 (9)
C8—C9—C10—C11	−0.9 (5)	C13—O3—C15'—C16	170.7 (6)
C8—C9—C10—Cl2	178.2 (3)	C15—O3—C15'—C16	61.3 (8)
C9—C10—C11—C12	−0.2 (5)	O3—C15—C16—C15'	68.7 (9)
Cl2—C10—C11—C12	−179.3 (2)	C14—C15—C16—C15'	−54.9 (11)
C8—C7—C12—C11	−2.0 (4)	C14—C15'—C16—C15	61.5 (10)
O1—C7—C12—C11	−179.1 (2)	O3—C15'—C16—C15	−60.7 (8)
C10—C11—C12—C7	1.6 (4)	C18—N1—C17—C13	83.7 (3)
C15—O3—C13—O2	−0.1 (6)	N2—N1—C17—C13	−94.0 (3)
C15'—O3—C13—O2	−32.6 (5)	O3—C13—C17—N1	62.6 (3)
C15—O3—C13—C17	−118.1 (6)	O2—C13—C17—N1	−53.3 (3)
C15'—O3—C13—C17	−150.5 (5)	C1—C13—C17—N1	−177.0 (2)
C15—O3—C13—C1	121.5 (5)	C19—N3—C18—N1	−0.2 (3)
C15'—O3—C13—C1	89.0 (5)	N2—N1—C18—N3	0.1 (3)
C14—O2—C13—O3	16.2 (3)	C17—N1—C18—N3	−177.8 (2)
C14—O2—C13—C17	133.3 (2)	N1—N2—C19—N3	−0.2 (4)
C14—O2—C13—C1	−104.1 (3)	C18—N3—C19—N2	0.2 (4)

### Hydrogen-bond geometry (Å, º)

**Table:**

*D*—H···*A*	*D*—H	H···*A*	*D*···*A*	*D*—H···*A*
C6—H6···O3^i^	0.95	2.48	3.314 (3)	146
C12—H12···O2^ii^	0.95	2.40	3.226 (3)	145
C17—H17*A*···O1^iii^	0.99	2.58	3.378 (3)	138
C18—H18···N3^iv^	0.95	2.57	3.323 (4)	136

Symmetry codes: (i) −*x*+1, −*y*, −*z*; (ii) −*x*+1, *y*, −*z*+1/2; (iii) −*x*+1, −*y*+1, −*z*; (iv) −*x*+1/2, −*y*+1/2, −*z*.
