# Supplementary figures and images for: Crystal structure of difenoconazole
Source: Acta Crystallogr Sect E Struct Rep Online. 2014 Oct 24;70(Pt 11):o1173. doi: 10.1107/S1600536814022429 (PMC4257337; doi:10.1107/S1600536814022429)

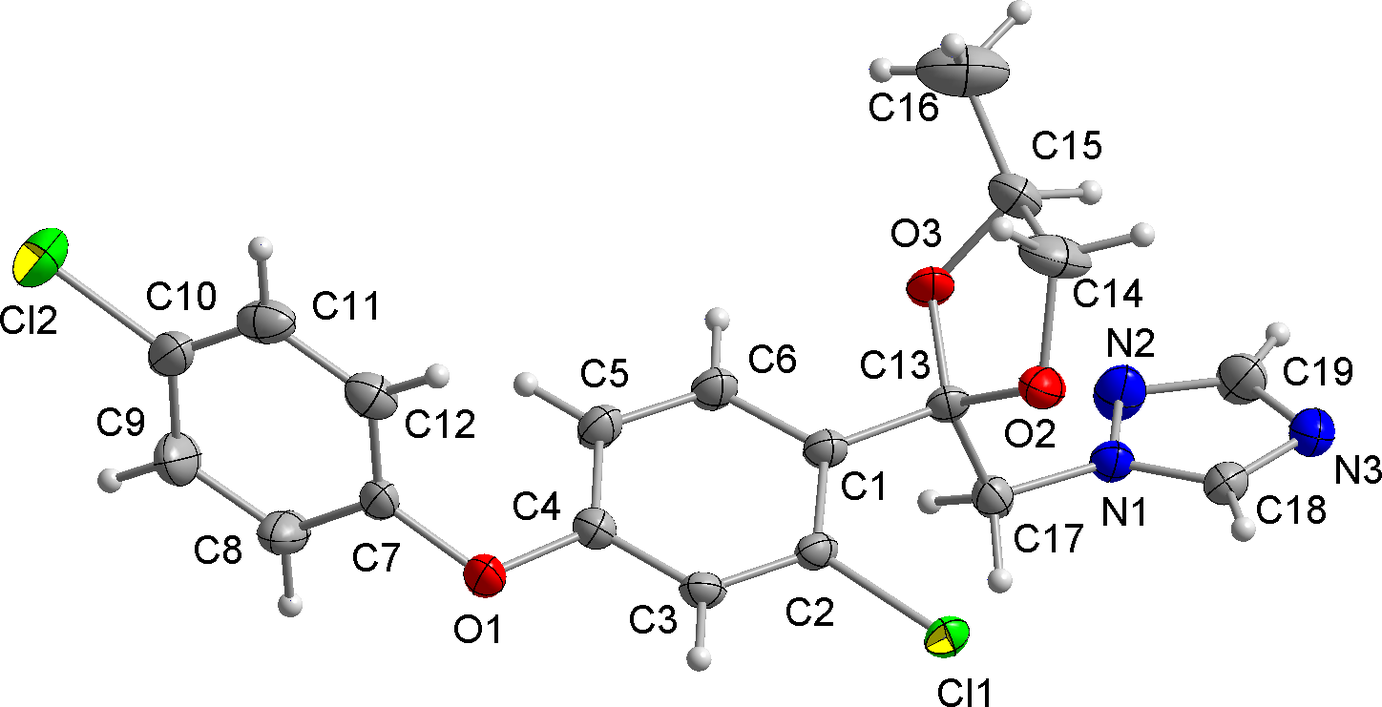

Supplement: Supplementary file 4 [file e-70-o1173-fig1.tif]

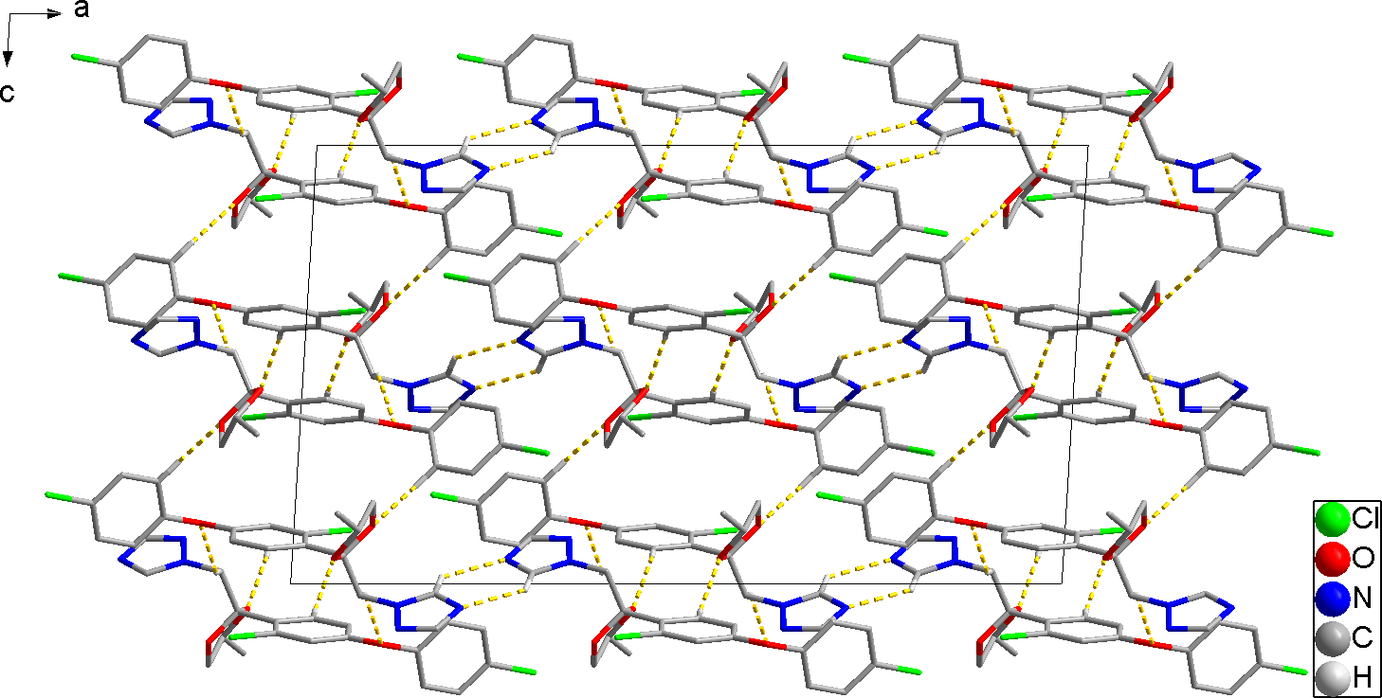

Supplement: Supplementary file 5 [file e-70-o1173-fig2.tif]
